# Treatment of Acute Orchitis by Puncturing the Testicle

**Published:** 1865-01

**Authors:** 


### TREATMENT OF ACUTE ORCHITIS BY PUNCTURING THE TESTICLE.

Henry Smith, Esq., Assistant-Surgeon King's College Hospital, in a case of very acute gonorrhœal orchitis, supposing supperation had taken place, with a view of evacuating the pus, made a free incision; but, to his dismay, no matter escaped. Speedy relief, however, followeed, and this case suggested some serious reflections. The quantity of serum and blood abstracted was too small, for the cessation of pain and swelling could hardly be due to this cause, and he was led to ascribe the relief to the removal of tension by the division of the fibrous envelope of the testis.

"Influenced," he says "by this reasoning, and by the result of this case, I determined to try the effect of puncturing the testis in similar cases; and in the next case of acute orchitis which presented, I made a deep and free incision with a sharp narrow bistoury, emitting about half a teaspoonful of serum and several drachms of blood; and no other treatment, beyond a little of the common aperient mixture, was supplied. The result here was as successful as in the former; and as cases presented themselves, I adopted the same plan of treatment, reserving it, however, especially to those instances where the swelling and pain were very great. After the trial in a few cases, it was found that the success attending this practice was such as to lead me to adopt it as the usual treatment of acute orchitis; and during the last twelve months I have probably treated in this way upwards of twenty cases, with results as have astonished both myself and those numerous pupils who have witnessed the practice.

"In nearly every case so treated—and I have purposely selected the most acute—the patient has experienced the most striking relief before he has left the out-patient's room; and on the next visit, forty-eight hours afterwards, the contrast presented is so remarkable that the superiority of this plan over the old-fashioned modes of treatment is at once impressed forcibly upon the minds of those even who would naturally be prejudiced against so apparently heroic a treatment. The speedy subsidence of all the acute symptons is due entirely to the puncture of the swollen and inflamed organ, for I have taken especial care not to prescribe anything else except a little of the common white mixture, or perhaps the use of the ordinary lead lotion, and this chiefly to please the patient.

"The only inconvenient result," he states, "I have witnessed from this treatment, was the following: An incision was made into the testicles of a middle aged man, with the usual relief, but in a few days the scrotum began to swell, great pain was experienced, and the man was taken into the hospital. The objectors to the mode of treatment suggested all sorts of disasters, in the shape of suppuration of the testicle, &c., but on careful examination it was ascertained that the swelling consisted of a large and rapid effusion of fluid into the tunica vaginalis, which was at once evacuated, with speedy relief to the patient. In another instance I made the incision much deeper than was necessary, carrying the point of the knife nearly to the back of the organ. As much as ten ounces of blood was lost, but the testis was violently inflamed and swollen, and the only effect of the accident was to make the patient somewhat faint, but at the same time to give more speedy and effectual relief than usual.

"This circumstance may lead one to the belief that the relief is due solely to the escape of blood from the puncture; bnt this view is inconsistent with the fact that great relief is given when only a few drachms of blood, mixed with serum, are discharged. Doubtless the direct withdrawal of blood from the highly inflamed testicle is of service, but my own view of the matter is, that the relief is in a great measure due to the withdrawal of the tension from the body of the testis by free division of the tunica albuginea."

In a postscript, Mr. S. adds:—"Since the above was written I have seen one of my old pupils who has been spending the last six months in the Paris hospitals, and he informs me that the ordinary practice at the Hopital de Midi, in cases of acute orchitis, is to make a puncture in several places with a lancet; the instrument is not carried into the body of the testicle, but simply through the tunica albuginea. He describes the plan of treatment as most successful."—*Amer. Jour. Med. Science, from the Lancet, Aug.* 6, 1864.

